# Targeting the FcεRI Pathway as a Potential Strategy to Prevent Food-Induced Anaphylaxis

**DOI:** 10.3389/fimmu.2020.614402

**Published:** 2020-12-17

**Authors:** Melanie C. Dispenza, Bruce S. Bochner, Donald W. MacGlashan

**Affiliations:** ^1^ Department of Medicine, Division of Allergy and Clinical Immunology, Johns Hopkins University School of Medicine, Baltimore, MD, United States; ^2^ Department of Medicine, Division of Allergy and Immunology, Northwestern University Feinberg School of Medicine, Chicago, IL, United States

**Keywords:** anaphylaxis, Bruton’s tyrosine kinase, Siglec, omalizumab, IgE, FcϵRI signaling, food allergy

## Abstract

Despite attempts to halt it, the prevalence of food allergy is increasing, and there is an unmet need for strategies to prevent morbidity and mortality from food-induced allergic reactions. There are no known medications that can prevent anaphylaxis, but several novel therapies show promise for the prevention of food-induced anaphylaxis through targeting of the high-affinity IgE receptor (FcϵRI) pathway. This pathway includes multiple candidate targets, including tyrosine kinases and the receptor itself. Small molecule inhibitors of essential kinases have rapid onset of action and transient efficacy, which may be beneficial for short-term use for immunotherapy buildup or desensitizations. Short courses of FDA-approved inhibitors of Bruton’s tyrosine kinase can eliminate IgE-mediated basophil activation and reduce food skin test size in allergic adults, and prevent IgE-mediated anaphylaxis in humanized mice. In contrast, biologics may provide longer-lasting protection, albeit with slower onset. Omalizumab is an anti-IgE antibody that sequesters IgE, thereby reducing FcϵRI expression on mast cells and basophils. As a monotherapy, it can increase the clinical threshold dose of food allergen, and when used as an adjunct for food immunotherapy, it decreases severe reactions during buildup phase. Finally, lirentelimab, an anti-Siglec-8 antibody currently in clinical trials, can prevent IgE-mediated anaphylaxis in mice through mast cell inhibition. This review discusses these and other emerging therapies as potential strategies for preventing food-induced anaphylaxis. In contrast to other food allergy treatments which largely focus on individual allergens, blockade of the FcϵRI pathway has the advantage of preventing clinical reactivity from any food.

## Introduction

Approximately 15 million people in the United States have food allergy and are at risk for anaphylaxis, a potentially life-threatening systemic allergic reaction (1). There is no cure for food allergy, and no known therapies are capable of preventing anaphylaxis, so food allergy is primarily managed by avoiding triggering foods. Unfortunately, accidental exposures still occur. In contrast, there are scenarios in which patients are intentionally exposed to known allergens such as during food oral immunotherapy (OIT). Despite its efficacy in inducing desensitization to protect against food-induced anaphylaxis, many patients stop OIT prior to reaching maintenance due to frequent and/or severe adverse reactions from the OIT doses themselves (2, 3). Therefore, there is an unmet need for novel strategies of preventing food-induced anaphylaxis from both accidental exposures and during therapeutic procedures such as OIT buildup.

Food allergy is mediated by food-specific IgE found in circulation and bound to the high affinity receptor FcϵRI on the surface of mast cells and basophils. When food protein binds to its specific IgE and cross-links the receptor, downstream activation of various kinases induces degranulation, leukotriene and prostaglandin production, and *de novo* cytokine production, which collectively cause clinical symptoms (4). Because all IgE-mediated reactions involve signaling through FcϵRI, targeting it or its pathway components would be an ideal strategy for preventing food-induced reactions. This review discusses components of the IgE pathway as potential therapeutic targets for preventing food-induced anaphylaxis (**Table 1**).

**Table 1:** Investigational therapies for food-induced anaphylaxis that target IgE or its pathway components.

Specific agents by target	Mechanism of action	Advantages	Disadvantages
IgE and FcϵRI			
Omalizumab* (anti-IgE mAb)	Prevents IgE binding to FcϵRI	Most well-studied potential therapy; favorable safety profile for chronic use; may facilitate food oral immunotherapy	Slow onset of action; incomplete prevention of clinical reactivity; most effective at lower serum IgE levels
Ligelizumab (anti-IgE mAb)	Prevents IgE binding to FcϵRI; may reduce B cell production of IgE	Higher affinity for IgE than omalizumab; favorable safety profile; may be more effective than omalizumab at high serum IgE levels	Moderately slow onset of action
Lumiliximab (anti-CD23)	Reduces B cell production of IgE	Long duration of total IgE reduction	Moderately slow onset of action
Quilizumab (anti-IgE mAb)	Depletes IgE-producing B cells	Long duration of total IgE reduction	Moderately slow onset of action
MEDI4212 (anti-IgE mAb)	Prevents IgE binding to FcϵRI; depletes IgE-producing B cells	May be more effective than omalizumab at high serum IgE levels	Moderately slow onset of action; shorter duration of action may mean more frequent dosing than omalizumab
E2_79 (anti-IgE darpin)	Prevents IgE binding to FcϵRI and dissociates existing complexes	Relatively rapid onset of action for dissociating IgE-FcϵRI complexes	No clinical data available
Kinases			
Ibrutinib, acalabrutinib (BTK inhibitors)	Inhibition of FcϵRI signaling	Complete inhibition of FcϵRI signaling; rapid onset of action; oral dosing; next-generation inhibitors have favorable safety profile; shown to prevent systemic anaphylaxis in humanized mice	Side-effects may prevent chronic use
Fostamatinib** (Syk inhibitor)	Inhibition of FcϵRI signaling	Rapid onset of action; oral dosing	Less favorable safety profile compared to other kinase inhibitors
GSK2646264 (Syk inhibitor)	Inhibition of FcϵRI signaling	Rapid onset of action	Currently in topical formulation only with unknown efficacy with oral dosing
WZ3146 (Lyn and Fyn inhibitor)	Inhibition of FcϵRI signaling	Rapid onset of action	No clinical data available; inhibitors may have increased toxicity due to broad enzyme expression
SHP and SHIP-1			
Anti-CD32b/FcϵRI mAb or fusion proteins	Inhibition of FcϵRI signaling		No clinical data available; risk of inducing anaphylaxis
Allergen-specific IgG	Inhibition of FcϵRI signaling; competitive binding of allergen away from IgE		Allergen-dependent mechanism
Anti-CD300a mAb IE1 (anti-CD300a/IgE bispecific Ab)	Inhibition of FcϵRI signaling		No clinical data available; only partial reduction of degranulation *in vitro*
AQX-1125	Inhibition of FcϵRI signaling	Oral dosing	Only partial reduction of degranulation *in vitro*
Siglecs			
Lirentelimab (AK002; anti-Siglec-8 mAb)	Inhibition of FcϵRI signaling	Target receptor has limited expression; favorable safety profile; shown to prevent systemic anaphylaxis in humanized mice; also depletes eosinophils	
Dual targeting liposomes (antigen and anti-Siglec-3/CD33)	Inhibition of FcϵRI signaling	Shown to prevent systemic anaphylaxis in mice	No clinical data available; allergen-dependent mechanism
Anti-Siglec-6 mAb	Inhibition of FcϵRI signaling	Target receptor is exclusive to mast cells	No clinical data available; only partial reduction of degranulation *in vitro*
Anti-Siglec-7 mAb	Inhibition of FcϵRI signaling		No clinical data available; less effective in preventing basophil activation *in vitro*

*FDA-approved for asthma and CSU.

**FDA-approved for chronic refractory immune thrombocytopenic purpura.

All agents act in an allergen-independent mechanism unless otherwise noted.

## IgE and FcεRI

IgE binding to FcϵRI stabilizes the receptor’s expression on the surface of mast cells and basophils; thus, FcϵRI expression is regulated by the level of total serum IgE (5, 6). Because the binding affinity of IgE to FcϵRI is extremely high (< 1 nmol/L), disruption of this interaction has proven difficult therapeutically. Strategies which focus on reducing the levels of total IgE or disrupting its binding to FcϵRI show promise for the prevention of IgE-mediated reactions to foods. The first demonstration that anti-IgE therapy could raise food threshold doses during oral challenge came from a trial with the anti-IgE monoclonal antibody (mAb) talizumab (developed by Tanox) in peanut-allergic subjects (7). Talizumab never progressed further in clinical trials, but other anti-IgE therapies show promise for this indication.

### Omalizumab

Omalizumab (Xolair^®^; manufacturers Genentech and Novartis) is a humanized antibody that binds to the C3 domain of IgE, thereby sequestering free IgE away from FcϵRI (8). The resulting downregulation of surface FcϵRI expression on mast cells and basophils renders them less sensitive to allergen-mediated activation. Already FDA-approved for asthma and chronic spontaneous urticaria (CSU), omalizumab has also demonstrated modest effect as a monotherapy for food allergy. Omalizumab treatment for 6–8 weeks increased patients’ threshold dose of peanut protein from a median of 80 to 6,500 mg in one open-label study in allergic adults (9), and another trial showed an 81-fold increase in peanut threshold dose in allergic subjects after 24 weeks of omalizumab compared to a 4.1-fold increase in the placebo-treated group (10). In food-allergic children receiving omalizumab for their severe asthma, omalizumab increased threshold doses for milk, egg, wheat, and hazelnut from an average of 1,013 to 8,727 mg of food protein after 16 weeks of treatment (11). Unfortunately, not all subjects in these trials displayed a significant increase in their food threshold dose on omalizumab. More research is needed to determine its efficacy in preventing reactivity from food exposures and to identify which patients would benefit most from omalizumab monotherapy.

Omalizumab has also been recently studied as an adjuvant therapy for food OIT. Early open-label studies suggested that 9 to 12 weeks of omalizumab therapy could facilitate rapid oral desensitization to cow’s milk and peanut in high-risk patients (12, 13). Subsequently, Wood et al. demonstrated in a double-blind, placebo-controlled (DBPC) trial that milk-allergic subjects treated with omalizumab experienced fewer adverse reactions during OIT build-up (with 2.1% of doses provoking reactions) compared to those treated with placebo (16.1% of doses) (14). None of the reactions experienced during omalizumab treatment required medical treatment, compared to 3.8% of reactions in subjects on placebo. However, omalizumab had no effect on subjects’ ability to pass an exit oral food challenge, nor did it affect rates of sustained unresponsiveness to milk 4 months after cessation of OIT. Another DBPC trial using omalizumab during multi-food OIT showed a significant reduction in the median per-participant percentage of OIT doses causing adverse reactions in those receiving omalizumab (27%) compared to placebo (68%), especially for gastrointestinal and respiratory symptoms (15). Additional trials are ongoing to evaluate omalizumab’s ability to facilitate multi-food OIT (NCT03881696).

### Ligelizumab

Ligelizumab (Novartis) is another humanized anti-IgE mAb which binds to free IgE with higher affinity than omalizumab. Ligelizumab effectively prevents passive systemic anaphylaxis (PSA) in human FcϵRIα transgenic mice (16). Interestingly, though ligelizumab also binds to the C3 domain of IgE, it can bind to CD23-bound IgE on B cells as well, unlike omalizumab (16). Though the mechanism is unclear, this property allows ligelizumab to prevent new IgE production. Ligelizumab has demonstrated superior and more durable suppression of total IgE levels, skin prick test responses to allergens, and basophil surface FcϵRI expression in humans compared to omalizumab (17). This may make it more effective than omalizumab in preventing IgE-mediated reactions in patients who have especially high serum IgE levels. It has not yet been tested in food allergy, though clinical trials have shown efficacy in CSU (NCT02477332).

### Lumiliximab

The low-affinity IgE receptor, FcϵRII or CD23, appears to regulate IgE homeostasis (18). Anti-CD23 treatment of B cells has been shown to reduce IgE production (19). In a phase I trial, a single dose of lumiliximab (Biogen Idec Inc.), an anti-CD23 antibody, significantly reduced circulating serum IgE in asthma patients (20).

### Quilizumab

Quilizumab (Genentech) is a humanized afucosylated anti-IgE antibody in development that binds to M1’, a domain specific to membrane-bound IgE in B cells. In this way, it reduces circulating IgE *via* apoptotic depletion of IgE-producing B cells, but does not bind to circulating IgE or IgE already bound to FcϵRI. In early clinical trials, a single dose of quilizumab reduced serum IgE levels in patients with allergic rhinitis or asthma for approximately 6 months (21). Unfortunately, despite reducing IgE levels, it failed to improve symptoms in a trial for antihistamine-refractory CSU (22). The future of quilizumab is unknown, but it may have a potential application in treating food allergy given its ability to prevent further IgE production.

### MEDI4212

MEDI4212 is a high-affinity antibody for IgE which can both bind to circulating IgE as well as membrane-bound IgE, thus depleting IgE-producing B cells through antibody-dependent cell-mediated cytotoxicity (23). Because of its dual action in reducing IgE, it may be more effective than omalizumab in patients with very high IgE levels. Its phase I trial demonstrated superior reduction of total IgE levels in atopic subjects after a single MEDI4212 dose compared to subjects treated with a dose of omalizumab (24).

### Anti-IgE Darpins

Eggel et al. created a non-immunoglobulin ankyrin repeat protein (DARPin) inhibitor of IgE, E2_79, which not only blocks IgE from binding to FcϵRI, but is also able to rapidly dissociate preformed IgE-FcϵRI complexes (25). Intravenous infusion of E2_79 just 6 hours prior to antigen challenge was shown to prevent passive cutaneous anaphylaxis (PCA) in human FcϵRIα transgenic mice (26). These data suggest that E2_79 could rapidly dissociate food-specific IgE from allergic cells in patients *in vivo*, though it has not yet been tested in clinical trials.

## Kinases

Numerous kinases are involved in FcϵRI pathway signaling, including spleen tyrosine kinase (Syk), Bruton’s tyrosine kinase (BTK), Lyn, Fyn, phospholipase Cγ (PLCγ), PI3 kinase (PI3K), and others (4). All of these enzymes are potential targets for inhibition of IgE-mediated activation of mast cells and basophils. Only recently, with the use of next-generation kinase inhibitors that are more specific for their target enzymes, have we begun to elucidate precisely which kinases are necessary to inhibit in order to prevent IgE-mediated reactions.

### BTK

BTK is largely expressed in leukocytes including mast cells, basophils, B cells, neutrophils, monocytes, and NK cells and is thought to be essential for IgE-dependent activation of human cells (27–29). As an essential enzyme for B cell receptor signaling, it has been pharmacologically targeted for the treatment of B cell malignancies, and the relatively recent FDA-approval of selective BTK inhibitors has created the opportunity to repurpose these medications for the prevention of IgE-mediated anaphylaxis. Dispenza et al. showed that just two clinically-relevant oral doses of acalabrutinib, a second-generation irreversible BTK inhibitor, completely prevented moderate-severity PSA in humanized NSG-SGM3 mice which have mature human mast cells and basophils (27). Even more remarkably, it significantly protected against mortality during fatal anaphylaxis in this mouse model. Data *in vivo* in humans is still preliminary, but ibrutinib has been shown to suppress IgE-mediated ex vivo basophil activation and skin prick testing to both aeroallergens and foods in allergic subjects (30–32). Clinical trials using BTK inhibitors to prevent clinically reactivity to foods in food allergic adults are currently ongoing in the authors’ laboratory.

### Syk

In addition to mast cells and basophils, Syk is expressed in numerous organ systems. Multiple studies have demonstrated Syk inhibition as a potential strategy for preventing anaphylaxis. The active metabolite of fostamatinib (Tavalisse^®^; Rigel Pharmaceuticals), which was approved in 2018 for chronic refractory immune thrombocytopenic purpura, prevented *ex vivo* basophil activation after a single dose in humans (33), as well as anaphylaxis to peanut in a mouse model of peanut allergy (34). GSK2646264 is a Syk inhibitor in a cream formulation in clinical trials for CSU (NCT02424799) which has been shown to attenuate IgE-mediated histamine release from human mast cells, though it is unclear if this compound would be safe and effective as an oral medication for the prevention of anaphylaxis (35). The Syk inhibitor NVP-QAB205 demonstrated excellent activity in preventing human mast cell and basophil activation (36, 37), but it did not progress to clinical trials due to potential toxicities like several other Syk inhibitors (38–41). Like BTK inhibitors, clinical trials are needed to demonstrate safety and efficacy for using Syk inhibitors to prevent food-induced anaphylaxis.

### Lyn and Fyn

Lyn and Fyn are kinases upstream of Syk in the FcϵRI pathway and may each play both positive and negative regulatory roles on IgE-mediated activation of human cells. Evidence that their inhibition can prevent IgE-mediated reactions largely arises from studies using non-specific compounds. The EGFR inhibitor WZ3146 effectively blocked mast cell and basophil activation *in vitro* through off-target antagonist activity on Lyn and Fyn (42). AZD7762, and inhibitor of Chk1 with Lyn/Fyn activity, and had similar efficacy in preventing LAD2 mast cell activation (43), but cardiac toxicity in Phase I trials prevented further development. Ultimately, Lyn and Fyn may be useful targets for preventing food allergy reactions if they can be specifically targeted, but even specific inhibitors may have an unfavorable toxicities given the broad expression profile of these kinases.

## SHP and SHIP-1

Upon activation, various mast cell and basophil inhibitory receptors with immunoreceptor tyrosine inhibitory motifs (ITIMs) recruit phosphotyrosine phosphatases (SHP‐1 and SHP‐2) and inositol phosphatases (SHIP‐1), which then provide direct inhibitory feedback on the FcϵRI pathway.

### CD32b

Expressed on basophils and some tissue mast cells as well as other cells, the low-affinity IgG receptor FcγRIIb (CD32b) is a potential target for the prevention of allergen-mediated anaphylaxis (44). Co-aggregation of CD32b with FcϵRI has been shown to inhibit IgE-mediated mast cell and basophil activation (45). Cross-linking these receptors can be achieved in an allergen-independent manner with bispecific antibodies to FcϵRI and CD32b or Fcϵ-Fcγ fusion proteins to prevent allergic reactivity (46–48), though these strategies have yet to move forward to clinical trials. In contrast, specific IgG antibodies can induce allergen-specific IgE-FcϵRI-CD32b cross-linking in the presence of allergen, as well as competitively block allergen binding to its specific IgE (49). Clinical trials using cat-specific IgG cocktails for treatment of respiratory allergies are ongoing (NCT03838731) (50), but no trials are currently investigating this approach in food allergy.

### CD300a

CD300a is an inhibitory receptor expressed on numerous human immune cells including mast cells and basophils. Cross-linking of CD300a recruits SHP-1 and SHIP-1 to elicit strong inhibitory signals on the FcϵRI pathway (51). Monoclonal antibodies to CD300a partially prevented IgE-mediated CD63 upregulation in human basophils (52, 53), and IE1, a bispecific antibody recognizing IgE and CD300a, was shown to inhibit FcϵRI signaling and IgE-mediated degranulation in human mast cells in a dose-dependent manner (54).

### AQX-1125

As a SHIP-1 activator, AQX-1125 reduced IgE-mediated mast cell degranulation *in vitro* and showed efficacy in murine models of allergic asthma (55). It is currently in clinical trials for atopic dermatitis (NCT02324972).

## Siglecs

Sialic acid-binding immunoglobulin-type lectins (Siglecs) are transmembrane receptors found on the surface of immune cells, so-called because they bind to sialic acid-containing ligands. Most Siglecs have ITIMs or ITIM-like motifs, which upon ligand binding, enable inhibitory signaling to counteract the actions of tyrosine kinases in the FcϵRI pathway (56, 57). Their differing ligand specificity and relatively restricted expression profiles make Siglecs good candidate targets for the suppression IgE-mediated activation of mast cells and/or basophils.

### Siglec-8

Siglec-8 is the best studied and most promising siglec target for the treatment of allergic diseases, with expression limited to human mast cells, basophils, and eosinophils (58, 59). *In vitro*, pre-incubation of human CD34-derived mast cells with anti-Siglec-8 mAb markedly shifted the anti-FcϵRI antibody-induced secretion dose response curve for histamine and PGD2 secretion (60). Siglec-8’s ITIM domain was found to be essential for this function, suggesting that its mechanism of action may involve phosphatase recruitment as discussed above. Furthermore, pretreatment with the humanized non-fucosylated IgG1 anti-Siglec-8 antibody lirentelimab (Allakos, Inc.) completely prevented human IgE-mediated PSA in NSG-SGM3 BLT humanized mice (61), suggesting that it may be a useful therapy for preventing anaphylaxis in humans. Lirentelimab has shown efficacy in other mast cell-driven diseases in early clinical trials, including CSU (NCT03436797), atopic keratoconjunctivitis (NCT03379311), and indolent systemic mastocytosis (NCT02808793). The most advanced efforts with lirentelimab involve a phase III study in eosinophilic gastritis and duodenitis (NCT04322604), a disorder characterized by increased numbers of tissue eosinophils and mast cells, based on positive phase II results (62).

### Siglec-3/CD33

CD33 (also known as Siglec-3) is expressed on most human myeloid cells, including mast cells and basophils. Duan et al. showed that dual targeting of CD33 and specific IgE on mast cells with liposomes expressing CD33L and a synthetic antigen (TNP) could prevent IgE-mediated anaphylaxis (63). These liposomes prevented TNP-induced degranulation in human mast cells and prevented clinical response during PSA in transgenic mice expressing human CD33. Intriguingly, the liposomes’ inhibitory effects were sustained for at least 2–3 days after infusion, potentially due to endocytosis of the TNP-IgE-FcϵRI complex in mast cells and/or liposomes’ interference of TNP binding to its specific IgE. One limitation of this approach is that these liposomes act in an antigen-specific manner, which may necessitate the use of several different types of liposomes to treat patients with multiple food allergies.

### Siglecs-6 and 7

Siglec-7 is expressed on human mast cells, monocytes, eosinophils, NK cells, and, to a lesser extent, basophils (64). Pre-incubation of CD34-derived mast cells with the combination of an activating anti-Siglec-7 antibody, an anti-IgE antibody, and a cross-linking anti-mouse IgG F(ab’)_2_ completely prevented degranulation and partially prevented PGD2 release and GM-CSF production (64). This inhibition was dependent on Siglec-7 directly crosslinking with FcϵRI, which has not always been found to be the case for other Siglecs that inhibit FcϵRI-mediated signaling such as Siglec-6 or -8 (60, 65). Interestingly, Siglec-7 engagement on human basophils only partially reduces IgE-mediated degranulation, a discrepancy thought to be due to the relatively low expression of Siglec-7 on basophils. Siglec-6 is a geographically unique target, given that its expression is primarily limited to mast cells (66), but its engagement on mast cells has shown only modest (approximately 30%) reduction in IgE-mediated degranulation (65). Further studies are needed to determine the efficacy of targeting either Siglec-6 or -7 for the prevention of IgE-mediated anaphylaxis.

## Discussion and Conclusions

Despite attempts at its prevention, the prevalence of IgE-mediated food allergy is increasing. Therapies targeting IgE and the FcϵRI pathway may fill the need for preventing food-induced anaphylaxis. One major benefit to this approach is that it is not allergen specific, unlike food OIT. Additionally, FcϵRI pathway inhibition would prevent the release of all mast cell and basophil mediators, unlike most current allergy treatments (such as antihistamines and leukotriene receptor antagonists) which only counteract the effects of a few of many mediators that participate in causing allergic reactions.

Both short-term and long-term protection strategies to prevent anaphylaxis are needed to improve the quality of life of food allergic patients. Short-term therapies which can facilitate food OIT build-up to prevent adverse reactions would allow more patients to reach maintenance dose. Alternatively, short-term treatments which could protect against accidental exposures during isolated high risk situations (e.g. a family vacation abroad or birthday parties) would reduce morbidity and mortality and help alleviate anxiety for those suffering from food allergies. Perhaps more importantly, therapies are needed which could be used chronically to reliably prevent reactions from accidental food exposures.

Many questions remain regarding the utility and safety of the above therapies, most of which are still experimental or in clinical development. Each therapy’s risk-benefit ratio and mechanism of action will determine its specific indication (**Table 1**). BTK inhibitors have a rapid onset of action and are highly effective at preventing IgE-mediated anaphylaxis, making them good candidates for short-term episodic use to prevent reactivity to foods, but they may not be suitable for long-term use based on the safety profile of the currently FDA-approved BTK inhibitors. Omalizumab has a favorable safety profile when used chronically, but it has slower onset of efficacy, and its ability to reliably prevent anaphylaxis (especially fatal anaphylaxis) is unknown. More studies are needed to determine the safety and efficacy of targeting the FcϵRI pathway as a protective measure against food-induced anaphylaxis.

## Author Contributions

MD conceived of the concept and wrote the manuscript. BB and DM contributed sections and provided feedback on the manuscript. All authors contributed to the article and approved the submitted version.

## Funding

This work was funded in part by a K23 mentored career award from NIH to MD (grant AI143965), a U19 grant from NIH to BB (AI136443), the Ludwig Family Foundation, and the Johns Hopkins School of Medicine Clinician Scientist Career Development Award.

## Conflict of Interest

MD has received research funding from Acerta Pharma/AstraZeneca in addition to consultant fees from AlphaSights, BVF Partners, and DAVA Oncology. BB has also received research funding from Acerta Pharma/AstraZeneca, as well as consultant fees from Regeneron, Sanofi, and GlaxoSmithkline. Finally, BB receives remuneration for serving on the scientific advisory board of Allakos, Inc. and owns stock in Allakos. He is a co-inventor on existing Siglec-8–related patents and thus may be entitled to a share of royalties received by Johns Hopkins University during development and potential sales of such products. BB is also a co-founder of Allakos; the terms of this arrangement are being managed by Johns Hopkins University and Northwestern University in accordance with their conflict of interest policies. DM consults for Boehringer-Ingelheim.
